# Antioxidant Properties of Fullerene Derivatives Depend on Their Chemical Structure: A Study of Two Fullerene Derivatives on HELFs

**DOI:** 10.1155/2019/4398695

**Published:** 2019-01-17

**Authors:** Vasilina Sergeeva, Olga Kraevaya, Elizaveta Ershova, Larisa Kameneva, Elena Malinovskaya, Olga Dolgikh, Marina Konkova, Iliya Voronov, Alexander Zhilenkov, Natalia Veiko, Pavel Troshin, Sergei Kutsev, Svetlana Kostyuk

**Affiliations:** ^1^Research Centre for Medical Genetics (RCMG), Moscow 115478, Russia; ^2^Institute of Problems of Chemical Physics of Russian Academy of Sciences, Moscow Region 142432, Russia; ^3^Skolkovo Institute of Science and Technology, Skolkovo Innovation Center, Nobel St. 3, Moscow, 143026, Russia

## Abstract

Oxidative stress is a major issue in a wide number of pathologies (neurodegenerative, cardiovascular, immune diseases, and cancer). Because of this, the search for new antioxidants is an important issue. One of the potential antioxidants that has been enthusiastically discussed in the past twenty years is fullerene and its derivatives. Although in aqueous solutions fullerene derivatives have shown to be antioxidants, their properties in this regard within the cells are controversially discussed. We have studied two different water-soluble fullerene C60 and C70 derivatives on human embryonic lung fibroblasts at a wide range of concentrations. Both of them cause a decrease in cellular ROS at short times of incubation (1 hour). Their prolonged action, however, is fundamentally different: derivative GI-761 causes secondary oxidative stress whereas derivative VI-419-P3K keeps ROS levels under control values. To gain a better understanding of this effect, we assessed factors that could play a role in the response of cells to fullerene derivatives. Increased ROS production occurred due to NOX4 upregulation by GI-761. Derivative VI-419-P3K activated the transcription of antioxidant master regulator NRF2 and caused its translocation to the nucleus. This data suggests that the antioxidant effect of fullerene derivatives depends on their chemical structure.

## 1. Introduction

Oxidative stress plays an important role in various diseases (Alzheimer's disease, schizophrenia, rheumatoid arthritis, diabetes, cardiovascular diseases, and cancer) and ageing [1–3]. Healthy cells produce physiological levels of reactive oxygen species (ROS) that can be localized in the cytoplasm, nucleus, or cell membrane. ROS is important for processes connected to cell cycle regulation, proliferation, apoptosis, and signaling, as well as kinase activity regulation and gene expression regulation [4]. Increased amounts of ROS lead to DNA damage, oxidative stress, and cell death [5–7]; therefore, novel antioxidants are of interest.

One of the synthetic antioxidants that gained a lot of attention in the past decades is fullerene and its derivatives. The chemical structure of fullerene derivatives allows them to effectively neutralize ROS [8]. Although it has been shown that fullerene derivatives act as strong antioxidants in solutions, the data concerning their antioxidant properties on cell cultures is controversial. Some studies confirm the antioxidative action of fullerenes whereas others show that cells treated with fullerenes exhibit signs of oxidative stress.

Fullerenes and their derivatives have shown cytoprotective action when cells were treated by ROS-inducing damaging factors. The polyvinylpyrrolidone fullerene C60 derivative registered as Radical Sponge® protects human keratinocytes from the damaging action of ultraviolet light that typically causes ROS production and oxidative stress [9]. In addition, fullerene C60 derivatives are able to neutralize TiO_2_-photocatalized ROS in keratinocytes and skin tissues [10]. Fullerenol C60(OH)36 is able to protect human erythrocytes against high-energy electrons [11].

However, the dependence of antioxidant action of fullerene derivatives on their chemical structure still remains unclear. It is believed that derivatives with a higher degree of functionalization have lower antioxidant activities due to disruption of the *π*-system. However, this generalization is not always correct, for example, malonic acid trisadducts proved to have a higher antioxidant activity than bisadducts containing ethylene glycol chains. It is unclear whether functionalization or the nature of the chemical moieties attached to the fullerene cage is more important for the antioxidant properties. In addition, fullerene derivatives are able to aggregate based on their chemical structure, thus decreasing their concentration and availability, as well as interact with enzymes and other biological molecules based on the structure and charge of the substance. This presents a challenge in discovering the dependence of antioxidant properties on the chemical structure of the fullerene derivative [12]. Antioxidant and prooxidant gene activation by fullerene derivatives remains an open question and requires further investigation. In this work, we have studied two water-soluble derivatives of fullerenes C60 and C70, bearing solubilizing residues of amino acid and aromatic acid on human embryonic lung fibroblasts (HELFs). Fullerene derivative GI-761 comprises five residues of 4-amino-3-phenylbutanoic acid and a chlorine atom arranged around one cyclopentadienyl unit on the [60]fullerene carbon sphere. On the contrary, solubilizing addends in the structure of compound VI-419-P3K are attached at the equator of the fullerene C70 cage. This study provides details and insights on the antioxidant activity of fullerene derivatives.

## 2. Materials and Methods

### 2.1. Cell Culture

Human embryonic lung cells (fourth passage) were provided by the Research Centre for Medical Genetics (RCMG). Approval#5 was obtained from the Committee for Medical and Health Research Ethics of RCMG. Cells were seeded at 1.7 × 104 per ml in DMEM (Paneco, Moscow, Russia) with 10% fetal calf serum (PAA, Vienna, Austria), 50 U/ml penicillin, 50 *μ*g/ml streptomycin, and 10 *μ*g/ml gentamycin, and cultured at 37°C for 2 or 24 h as previously described in [13, 14]. Investigated fullerene derivatives were added to the medium, and the cells were cultured for periods ranging from 15 min to 48 h.

### 2.2. Antibodies

Primary antibodies FITC-*γ*H2AX (pSer139) (Chemicon, Temecula, USA), NRF2, BRCA2 (Santa Cruz Biotechnology, Dallas, USA), NOX4, pNRF2, and FITC goat anti-rabbit IgG (Abcam, Cambridge, UK) were used.

### 2.3. Flow Cytometry Analysis (FCA)

Cells were washed with Versene solution, then treated with 0.25% trypsin, washed with culture medium, and suspended in PBS. Paraformaldehyde (PFA, Sigma-Aldrich, Saint Louis, USA) treatment at 37°C for 10 min was performed to fix the cells. Cells were washed three times with 0.5% BSA-PBS and permeabilized with 0.1% Triton X-100 in PBS for 15 min at 20°C or with 90% methanol at 4°C, then washed with 0.5% BSA-PBS (3 times) and stained with antibodies (1 *μ*g/ml) for 2 h at 4°C, and washed three times with 0.5% BSA-PBS. The cells were then incubated for 2 h (20°C) with FITC goat anti-rabbit IgG (1 *μ*g/ml) and analyzed.

### 2.4. Fluorescence Microscopy

The Axio Scope.A1 microscope (Carl Zeiss) and the confocal microscopy platform Leica TCS SP8 (Germany) were used for fluorescent microscopy of stained cells.

### 2.5. Immunocytochemistry

Cells were grown in slide flasks (25 cm^3^, Thermo Fisher Scientific, Waltham, USA), fixed in 3% paraformaldehyde at 4°C for 20 min, washed with PBS, and then permeabilized with 0.1% Triton X-100 in PBS for 15 min at room temperature, followed by blocking with 0.5% BSA in PBS for 1 h and incubation overnight at 4°C with the antibodies. After washing with 0.1% Triton X-100 in PBS, fibroblasts were incubated for 2 h at room temperature with the FITC goat anti-mouse IgG, washed with PBS, and then stained with DAPI as described in [13, 14].

### 2.6. Reactive Oxygen Species (ROS) Assays

Cells were grown in 96-well plates, incubated with investigated derivatives, washed with PBS, and treated with 10 *μ*M solution of H2DCFH-DA in PBS (Molecular Probes/Invitrogen, Carlsbad, USA) for 20 min. Сells were washed three times with PBS and analyzed at 37°C using the total fluorescence assay in a plate reader at *λ*ex = 488 nm and *λ*em = 528 nm (EnSpire Equipment, Turku, Finland). The ROS analysis was performed with three techniques: flow cytometry, fluorescent microscopy, and total fluorescence assay in a 96-well plate. The reaction rate constant for the formation of DCF (*k*) was calculated using the dependence of the DCF signal intensity on the time of cell incubation with H2DCFH-DA. The data are presented as the *k*_*i*_/*k*_0_ ratio, where *k*_*i*_ and *k*_0_ are the rate constants in the exposed and unexposed cells, respectively. The average value of the DCF signal for 16 wells ± standard deviation is reported.

### 2.7. MTT Assay

Cells were grown in a 96-well plate for 72 h. Survival was measured using the 3-(4,5-dimethylthiazol-2-yl)-2,5-diphenyltetrazolium bromide (MTT) assay, as described previously [13, 14]. The plates were read at 550 nm (EnSpire reader).

### 2.8. Quantification of mRNA Levels

Total mRNA was isolated using the RNeasy Mini Kit (Qiagen, Hilden, Germany), treated with DNAse I, and then reverse transcribed by the Reverse Transcriptase kit (Sileks, Moscow, Russia). The expression profiles were obtained using qRT-PCR with SYBR Green PCR Master Mix (Applied Biosystems, Foster City, USA). The mRNA levels were analyzed using StepOnePlus (Applied Biosystems); the technical error was approximately 2%. *TBP* was used as a reference gene. The following primers were used (Sintol, Moscow, Russia): *BRCA1* (F: TGTGAGGCACCTGTGGTGA, R: CAGCTCCTGGCACTGGTAGAG); *NRF2* (*NFE2L2*) (F: TCCAGTCAGAAACCAGTGGAT, R: GAATGTCTGCGCCAAAAGCTG); *NOX4* (F: TTGGGGCTAGGATTGTGTCTA; R: GAGTGTTCGGCACATGGGTA); *BRCA2* (F: CCTCTGCCCTTATCATCACTTT; R: CCAGATGATGTCTTCTCCATCC); *CCND1* (F: TTCGTGGCCTCTAAGATGAAGG; R: GAGCAGCTCCATTTGCAGC); and *TBP* (reference gene) (F: GCCCGAAACGCCGAATAT, R: CCGTGGTTCGTGGCTCTCT).

### 2.9. Comet Assay

Cells were suspended in low-melting-point agarose and placed onto slides precoated with 1% normal-melting-point agarose. The slides were placed in a solution of 10 mM Tris-HCl (pH 10), 2.5 M NaCl, 100 mM EDTA, 1% Triton X-100, and 10% DMSO at 4°C for 1 h. Electrophoresis was performed for 20 min at 1 V/cm and 300 mA in an electrophoresis buffer (300 mM NaOH, 1 mM EDTA, and pH > 13). The slides were fixed in 70% ethanol and stained with SYBR Green I (Invitrogen, USA).

Images of comets were analyzed using CASP v.1.2.2 software.

### 2.10. Statistics

The results were repeated at least three times as independent biological replicates. In FCA, the medians of the signal intensities were analyzed. The figures show the mean and standard deviation (SD). The significance of the observed differences was analyzed with nonparametric Mann-Whitney *U* tests. *P* values < 0.05 were considered statistically significant and marked on figures with “∗.” Data were analyzed with StatPlus2007 professional software (http://www.analystsoft.com).

### 2.11. Synthesis of the Fullerene Derivatives

**Figure 1:**
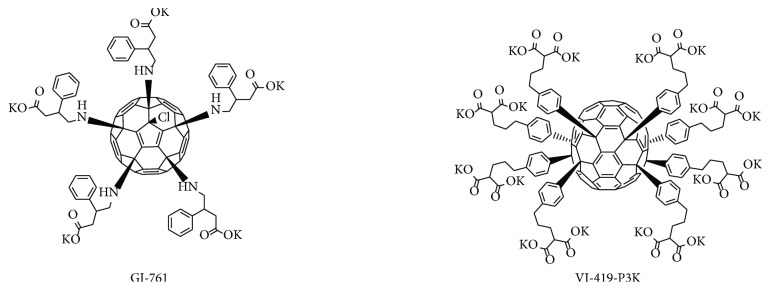
Molecular structures of the investigated water-soluble fullerene derivatives GI-761 and VI-419-P3K.

Polycarboxylic water-soluble fullerene derivatives GI-761 and VI-419-P3K (Figure 1) were synthetized in three steps from the readily available precursors C_60_Cl_6_ and C_70_Cl_8_ [15] following previously developed methodology [16, 17].

Details of the synthesis of the compounds and their spectral characterization data are provided in the supporting information. Both compounds showed high solubility in water and culture medium in the presence as well as in the absence of serum.

## 3. Results and Discussion

Investigated derivatives were prescreened for toxicity towards HELFs by the MTT assay as described in [13, 14]. When the concentration of the used derivatives was higher than 20 *μ*M, at least 50% of the treated cells died within 3 days.

### 3.1. GI-761 and VI-419-P3K Affect ROS Production in HELFs

**Figure 2:**
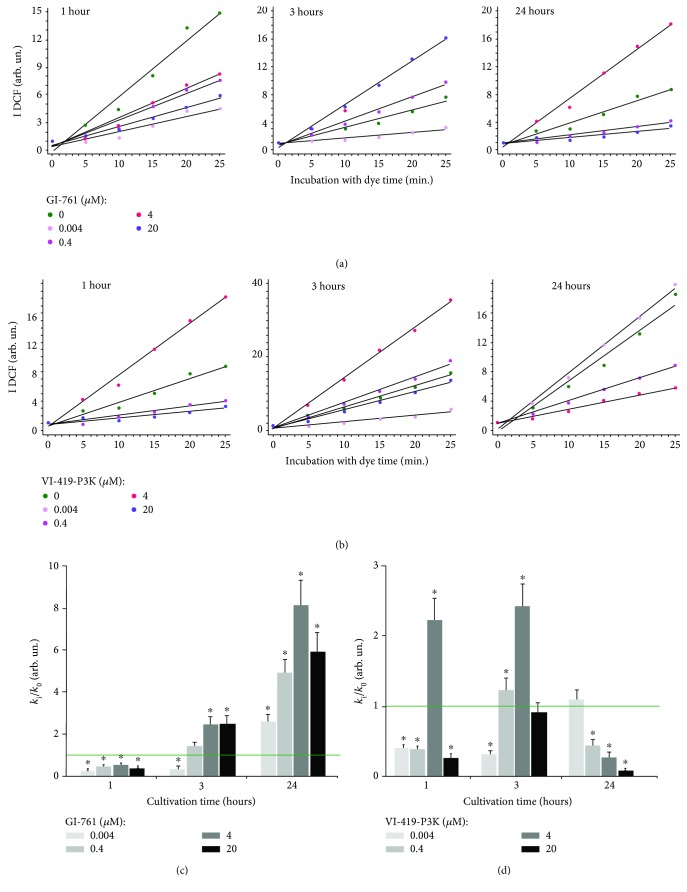
ROS levels in cells treated with fullerene derivatives. (a, b) Fluorescence plate reader: dependence of DCF signal intensity on GI-761 (a) and VI-419-P3K (b) concentration and exposure time. (c, d) Dependence of the *k*_*i*_/*k*_0_ index on GI-761 (c) and VI-419-P3K (d) concentration and exposure time.

ROS production level was measured with the DCF reagent in a plate reader (EnSpire, Finland). The dependence of DCF fluorescence on time is shown in Figure 2. The inclination (*k*) reflects the ROS production rate. Linear approximation was used to calculate the inclination. The DCF synthesis rate shows the amount of ROS in the cell culture. The results are shown as a ratio of *k*_*i*_/*k*_0_, where *k*_*i*_ is the inclination for the time and concentration of interest and *k*_0_ is the inclination in control cells.

Initially (1 h), GI-761 at both low (4 nM and 0.4 *μ*M) and high (4 *μ*M and 20 *μ*M) concentrations significantly reduces the amount of ROS in the cells. In 3 h, the amount of ROS in cells in the presence of 0.4, 4, and 20 *μ*M of fullerene increases. The concentration of 4 nM of GI-761 keeps ROS levels below the control level. GI-761 caused a significant increase in ROS levels at all investigated concentrations (4 nM, 0.4, 4, and 20 *μ*M) after 24 h of incubation with HELFs.

Derivative VI-419-P3K at low (4 nM and 0.4 *μ*M) and high (20 *μ*M) concentrations significantly reduces the amount of ROS in the cells after 1 h of incubation. The concentration of 4 *μ*M, however, stimulates the synthesis of ROS. ROS production increases after 3 h of incubation with 0.4 and 20 *μ*M of VI-419-P3K. At a concentration of 4 nM, the ROS levels do not change. At a concentration of 4 *μ*M of fullerene, VI-419-P3K stimulates ROS synthesis to the greatest extent. After a day of incubation with VI-419-P3K (0.4, 4, and 20 *μ*M), a significant decrease in ROS levels is observed. The ROS levels were increased in the presence of 4 nM of fullerene, but did not exceed the control level.

**Figure 3:**
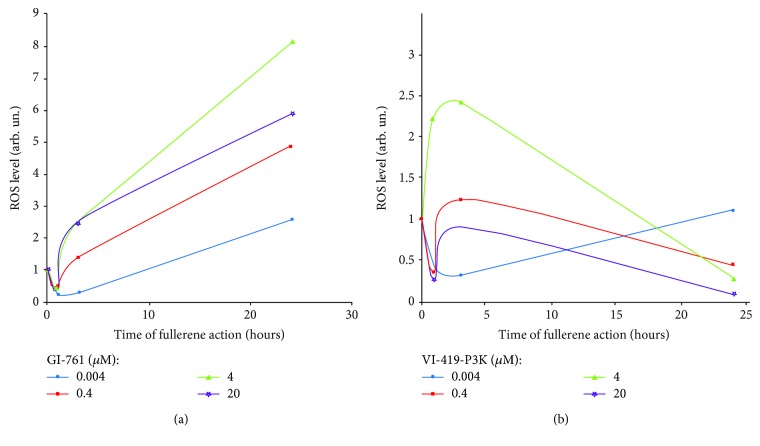
Dependence of ROS levels in HELFs treated with GI-761 (a) and VI-419-P3K (b) on time of incubation.

The scheme illustrating the change of ROS levels in fibroblasts under the action of fullerenes GI-761 and VI-419-P3K is shown in Figure 3. All studied concentrations of GI-761 cause rapid decrease of ROS levels in cells 1 h after addition, but in 3 h, it is replaced by increasing ROS levels (Figure 3(a)). The maximum increase in ROS levels is observed after 24 hours of cultivation at a concentration of 4 *μ*M. At very low concentrations (4 nM), VI-419-P3K causes a rapid decrease in ROS levels in cells, which lasts at least three hours and returns to the control values after 24 hours of cultivation (Figure 3(b)). At a low concentration (0.4 *μ*M), ROS levels drop after 1 hour, then after 3 hours, increase above the control, and after 24 hours, decrease again. At a concentration of 4 *μ*M, VI-419-P3K causes a significant increase in ROS levels after 1-3 hours of exposure. In 24 hours of incubation with VI-419-P3K, the synthesis of ROS was below the control values. High concentration of fullerene induced a rapid decrease in ROS levels after 1 hour, a return to the control level after 3 hours, and again a very sharp decrease after 24 hours.

Nonlinear dependence of the ROS changes indicates a complex nature of fullerenes' effect on ROS levels in cells. It is possible that ROS levels in cells are affected by two countervailing processes. The first process is the binding of ROS by nanoparticles of fullerenes (a similar effect for carbon-based nanomaterials is shown in [18]). However, in response to the primary reduction of ROS, processes aimed at ROS production are rapidly activated. The resulting effect is determined by the concentration of fullerenes and the time of action. To determine what is the cause of the detected nonlinear dependence of the cell response to GI-761 and VI-419-P3K, several cellular parameters that can affect ROS levels were analyzed.

**Figure 4:**
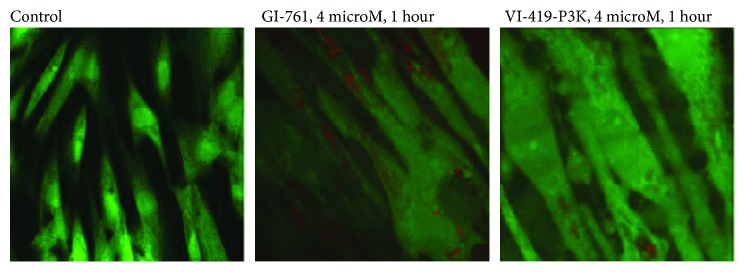
Colocalization of fullerene derivatives GI-761 and VI-419-P3K (red stains) and ROS stained with DCF, fluorescent microscopy.

Colocalization of investigated fullerene derivatives and ROS (stained with DCF) is shown in Figure 4. Red stains of fullerene derivatives are surrounded by black spots where there is no ROS indicating that the derivatives neutralize ROS in their surroundings.

### 3.2. GI-761 and VI-419-P3K Affect Prooxidant and Antioxidant Gene Expressions in HELFs

One of the main producers of physiological amounts of ROS in cells is NADPH oxidases [19] (NOX family). We have previously shown that ROS production in cells treated with fullerene derivatives increased due to NOX4 activation [13]. We measured the amount of NOX4 using the method of flow cytometry.

**Figure 5:**
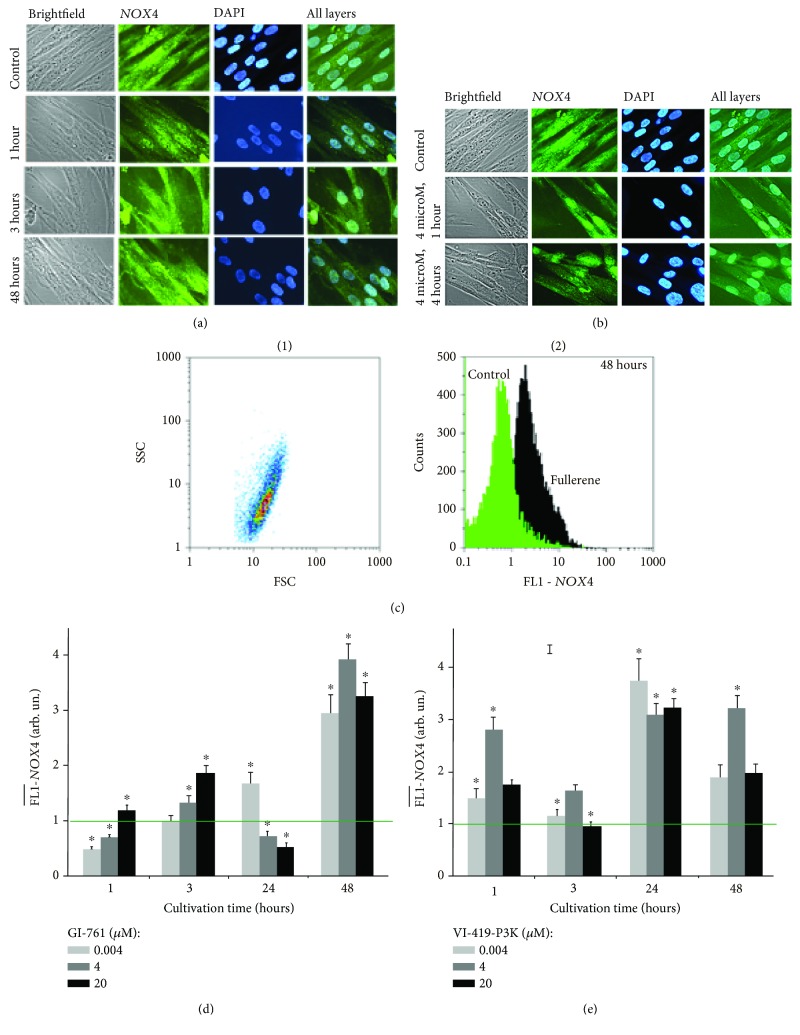
NOX4 expression in HELFs incubated with GI-761 and VI-419-P3K. (a, b) Fluorescent microscopy: localization of NOX4 within fixed cells stained with DAPI and antibodies to NOX4 in HELFs treated with (a) GI-761 and (b) VI-419-P3K. (d, e) FCA: FL1-NOX4 levels for HELFs treated with GI-761 (d) and VI-419-P3K (e). Concentrations and times of exposure shown in graphs.

Average signals of FL1-NOX4 per cell are shown in Figures 5(d) and 5(e). After 1 hour of cultivation with GI-761 at concentrations of 4 nM or 4 *μ*M, a decrease in the expression of NOX4 protein was detected. At a concentration of 20 *μ*M, GI-761 increases the level of NOX4. In 3 h, GI-761 causes an increase in the amount of NOX4 protein levels at all studied concentrations. Maximal amount of NOX4 was seen at 20 *μ*M of fullerene. Longer cultivation (24 hours) is accompanied by a repeated decline in the amount of NOX4 in fibroblasts at 4 and 20 *μ*M concentrations of fullerene derivative GI-761. In 48 hours, an increased level of protein expression was observed for all used concentrations of fullerenes.

One hour after the addition of the compound to the culture medium of HELFs at a concentration of 4 *μ*M, VI-419-P3K fullerene derivative causes an increase in the level of NOX4 in cells. 4 nM and 20 *μ*M reduce the amount of NOX4. In 3 hours, a decrease in the amount of NOX4 is observed for all studied concentrations of VI-419-P3K. Maximal amounts of NOX4 in HELFs were at 4 *μ*M of fullerene. Longer cultivation (24 hours) is accompanied by a repeated increase in the NOX4 levels in fibroblasts at all tested concentrations of fullerene VI-419-P3K. In 48 hours, an increased level of the protein expression was observed only for 4 *μ*M fullerene VI-419-P3K.

Thus, both ROS levels and NOX4 protein levels have a nonlinear dependence on the time of incubation and the concentration of the studied substances.

**Figure 6:**
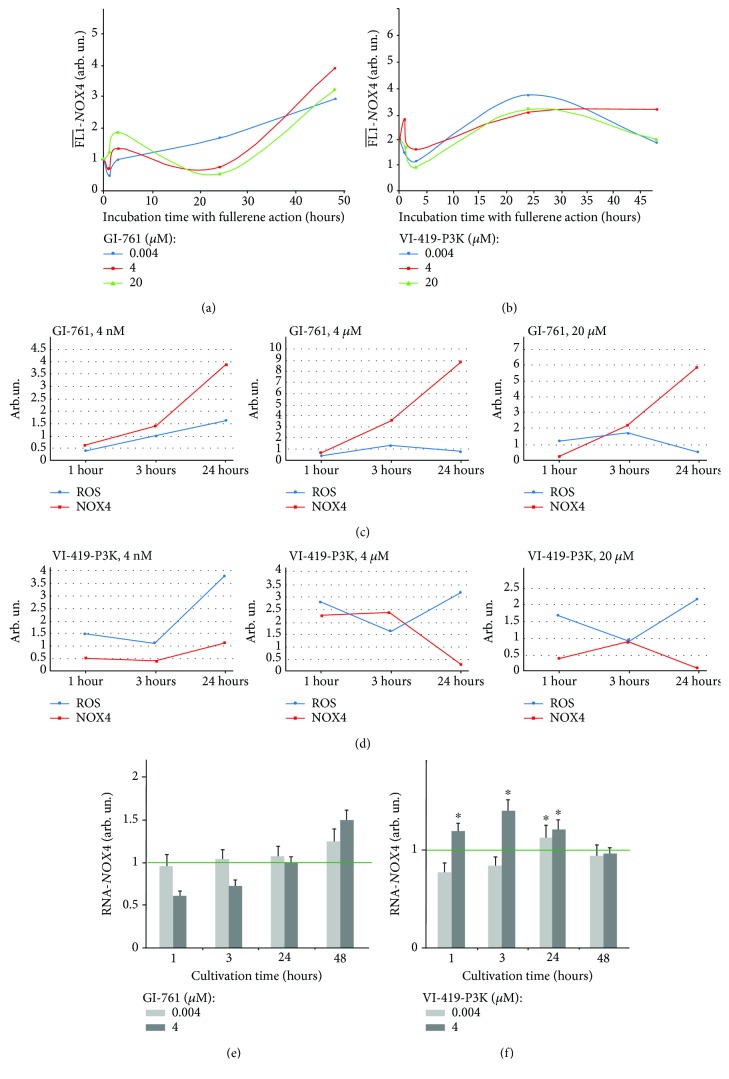
Dependence of NOX4 protein levels on time for HELFs treated with GI-761 (a) and VI-419-P3K (b). Concentrations and times of exposure shown in graphs. (c, d) Dependence of NOX4 protein levels and ROS levels shown in one graph. NOX4 mRNA levels in HELFs treated with GI-761 (e) and VI-419-P3K (f).

The gene expression of NOX4 on the mRNA levels was assessed with quantitative real-time PCR. The data is shown in Figure 6. Data concerning the amount of NOX4 mRNA in cells treated with 4 nM of GI-761 and VI-419-P3K fullerenes correlate with the protein NOX4 data. Thus, we can say that regulation of the gene expression of NOX4 in response to the action of 4 nM fullerene is regulated mainly on the level of gene transcription. The amount of protein in the case of 4 *μ*M of GI-761 is different from the amount of mRNA. Despite the same amount of mRNA of a gene, the amount of protein in the case of 4 *μ*M fullerene was much higher than that in the case of 4 nM. Another exception was the data for VI-419-P3K after 48 h of cultivation. Apparently, under certain conditions, fullerenes can regulate amounts of protein on a posttranscriptional level.

As NOX4 catalyzes the formation of ROS, it is important to know where the enzyme is located in the cell. The accumulation of the enzyme close to the nucleus or in the nucleus itself can contribute to enhance the synthesis of ROS there and oxidative DNA damage. The localization of NOX4 in cells treated with fullerene derivatives was assessed with fluorescent microscopy (Figures 5(a) and 5(b)).

In the control cells, NOX4 is localized mainly in the cytoplasm and near the plasma membrane of cells. Small amounts of protein can be found in the nuclei of some cells. Concentrations of 4 nM, 4 *μ*M, and 20 *μ*M of fullerenes GI-761 and VI-419-P3K had a similar effect on the change of NOX4 localization depending on time. The most significant change was observed in the case of 4 *μ*M of fullerene. Fullerene stimulates the expression of NOX4 in the nuclei of the cells. After 1 hour, the NOX4 signal is clearly visible in the nucleus compared to the cytoplasm of cells. In 3 h, NOX4 is still present in the cell nucleus but is also increased in other structures of the cell. After prolonged cultivation (48 hours), the level of NOX4 expression reduced in the nuclei of cells and is increased in the cytoplasm of cells.

Short-term cultivation of HELFs with fullerene increases the amount of NOX4 in the nuclei of the cells, and this should lead to increased synthesis of ROS in the nuclei of the cells. Localization of ROS in nonfixed cells treated with 4 *μ*M of fullerene for 1 hour was assessed in order to confirm the immunofluorescence data. The data is shown in Figure 5.

In control cells, the ROS synthesis occurs in the cytoplasm, mainly in the mitochondria (meandering bright lines) and on the surface of the cell membrane (bright spots, remote from the nuclei). In the presence of fullerenes GI-761 and VI-419-P3K, the main signal of DCF is visible in the cell nucleus. This change in the localization of ROS in the cell may be responsible for the apparent small decrease in ROS levels.

We analyzed the dependence of ROS levels on the amounts of NOX4 in the treated cells.  Figure 6 shows the dependence for three time points and three different concentrations of fullerenes GI-761 and VI-419-P3K.

GI-761 causes an increase in the NOX4 expression after 3 h of incubation. This is accompanied by an increase in ROS levels in the cells. In 24 h, despite a decrease in NOX4, increased levels of ROS are present in the cells. VI-419-P3K at concentrations of 4 *μ*M and 20 *μ*M increases the NOX4 expression in 1 h of incubation, which leads to an increased ROS amount in 3 h. In 24 h, however, the NOX4 expression is increased, but the ROS levels are decreased.

This response can be divided into two stages: early (1-3 hours) and late (24-48 hours) stages. After 1 hour of cultivation with GI-761 (4 nM-4 *μ*M) and VI-419-P3K (4 nM-20 *μ*M), there is a decline in the physiological level of ROS in cells, which indicates the ability of fullerenes GI-761 and VI-419-P3K to effectively neutralize ROS and the possibility of their use as antioxidants (with the exception of 4 *μ*M for VI-419-P3K). However, the “late” response of cells to the fullerenes GI-761 and VI-419-P3K is different—VI-419-P3K has a prolonged (up to 24 hours) antioxidant effect, whereas fullerene GI-761 causes a secondary peak in the ROS synthesis after 24 hours of incubation.

Thus, the effect of fullerene derivatives on the ROS production in HELFs depends on the chemical moieties attached to the fullerene core and on the shape and size of the fullerene cage. To assess the possible cause of time dependence and to explain the difference between the actions of the two investigated fullerene derivatives on HELFs, we looked at the antioxidant response system of the cells.

**Figure 7:**
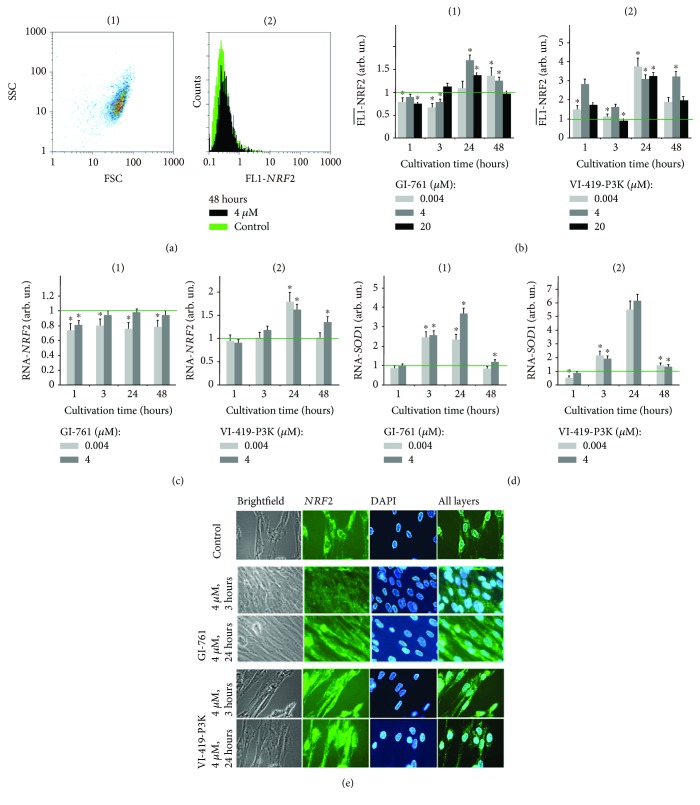
NRF2 levels in HELFs treated with investigated substances. (b) FCA: levels of FL1-NRF2 in cells treated with GI-761 and VI-419-P3K. (c) Real-time PCR: levels of NRF2 mRNA in cells treated with GI-761 and VI-419-P3K. (e) Fluorescent microscopy: localization of NRF2 protein in cells treated with GI-761 and VI-419-P3K, nuclei of the cells stained with DAPI.

NRF2 (erythroid-derived factor 2) is one of the main transcription factors that determine antioxidant response of the cells to the action of the internal and external ROS. NRF2 controls constitutive inducible expressions of several genes that contain ARE in their promotor region [20]. NRF2 is regulated both at the level of amount of protein and at the level of its localization in the cells as the factor only affects the gene expression within the nucleus [21]. It is known that NRF2 regulates ROS production by the mitochondria and NADPH oxidases [22]. The change in the NOX4 expression and ROS levels suggests that the activity of NRF2 can be altered in the presence of fullerenes. The amount of NRF2 in the cells was assessed with flow cytometry (Figure 7); an average signal intensity of FL1-NRF2 per cell was detected.

Within the first 3 h of incubation, GI-761 (4 nM, 4, and 20 *μ*M) causes no increase in the NRF2 expression in HELFs, but in 24-48 h, a slight increase (1.2–1.7-fold) is detected. Unlike GI-761, derivative VI-419-P3K induces the activation of NRF2. After 1 h of incubation of VI-419-P3K (4 nM, 4 *μ*M, and 20 *μ*M), an increase of NRF2 (1.5, 2.8, and 1.8 times, respectively) is observed (Figure 7(b)). After 3 h with 4 nM of fullerene, the amount of NRF2 is reduced. After 24 h of incubation with all investigated concentrations, VI-419-P3K caused an increase in the NRF2 levels 3-4-fold (Figure 7(b)).

The change in the NRF2 mRNA amount under the action of fullerenes is shown in Figure 7(c). GI-761 does not affect the level of NRF2 gene expression at all investigated concentrations and times of incubation. These data indicate that GI-761 can regulate the expression of the transcription factor NRF2 at both the transcription level and the posttranscriptional level. Most likely, fullerene can promote the release of NRF2 from the complex with its inhibitor KEAP1 [23], resulting in a better recognition of free NRF2 by antibodies.

Unlike GI-761, derivative VI-419-P3K causes the activation of NRF2 gene expression in 24 and 48 hours, which correlates with the increased levels of NRF2 protein (Figure 7(b)). Increase in the NRF2 protein level 1 hour after addition of VI-419-P3K is likely to be explained with the release of NRF2 from its complex with KEAP1.

The determining factor in the activity of NRF2 as a transcription factor is its localization in the cell. Cells treated with fullerenes were studied using fluorescence microscopy. There was no translocation of NRF2 to the nucleus of HELFs after 3 h of incubation with any of the studied concentrations of GI-761 (Figure 7(e)). On the contrary, at 4 *μ*M of GI-761, the factor NRF2 was migrating from the nuclei of those cells where it was present in the nucleus, to the cytoplasm. Even though after 24 hours of incubation with GI-761 the NRF2 amount increases in the cytoplasm, NRF2 does not translocate to the nucleus. Thus, fullerene GI-761 appears to be blocking the activity of NRF2, despite the increase of the factor at some concentrations.

All investigated concentrations of VI-419-P3K cause an increase in the NRF2 expression within 3 h of incubation, but NRF2 does not translocate to the nucleus. In 24 h, the NRF2 expression increases both in the cytoplasm and the nucleus. Thus, derivative VI-419-P3K causes the activation of the transcription factor NRF2 both on the gene and the protein level and the translocation of the factor to the nucleus. Most likely, the prolonged antioxidant action of VI-419-P3K can be explained with NRF2 activation.

### 3.3. DNA Breaks

**Figure 8:**
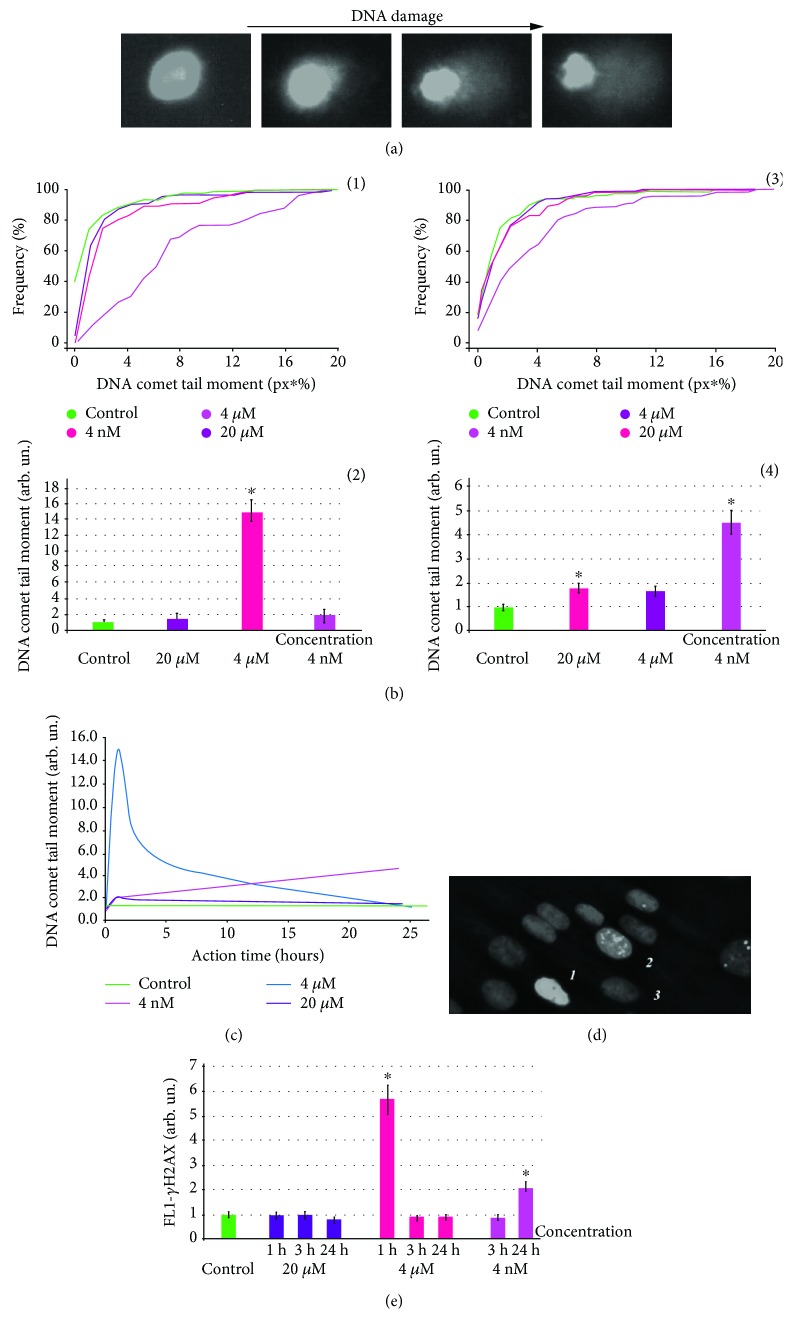
DNA damage in cells incubated with GI-761 and VI-419-P3K. (a–c) Comet assay: (a) digital photography of nuclei with varying degree of DNA damage; (b) dependence of “% of tail DNA” on fullerene derivative concentration; (c) dependence of DNA comet tail moment on the time of incubation with fullerene derivatives. (d) Fluorescent microscopy: cells stained with antibodies for H2AX. (e) FCA: average signal intensity of FL1-*γ*H2AX given for times and concentrations of investigated fullerene derivatives.

Since investigated substances affect ROS production in HELFs and increased levels of ROS usually lead to DNA damage [24], we investigated the level of DNA breaks in the treated cells. As the addition of GI-761 to HELFs caused increased ROS levels already at a concentration of 4 *μ*M, the level of DNA breaks after 24 h of incubation when GI-761 (4 nM) was assessed. In 24 hours, fullerene GI-761 at a concentration of 4 nM induces a 4.6-fold increase in the amount of DNA breaks as was shown by single cell electrophoresis (comet assay) (Figure 8(a)). This fact correlates with the early ROS production in the treated cells. To confirm this result, we used the gamma-foci method. Gamma-foci is a well-known method of double-strand DNA break detection that is based on the antibody labelling of a phosphorylated form of histone protein H2AX. This form appears at the double-strand DNA break sites [25]. Medians of H2AX levels in the cells obtained by flow cytometry confirmed single cell electrophoresis data. 4 nM of GI-761 increases the amount of double-strand DNA breaks in treated HELFs after 24 hours of incubation (Figures 8(d) and 8(e)).

In 48 h, the amount of double-strand DNA breaks returned to control levels. This could be either due to activation of the DNA reparation system or due to the death of cells with damaged DNA. As we did not detect increased levels of cell death in the population, we assessed the activation of the reparation systems in treated HELFs. Two main genes that are activated in the DNA reparation are *BRCA1* and *BRCA2*. 4 nM of GI-761 increased the expression of the *BRCA1* and *BRCA2* genes 1.5-fold after 24 h of incubation. These data show that cells effectively respond to the DNA breaks induced by GI-761. Activation of DNA reparation systems is usually accompanied by cell cycle arrest.

Cyclin D1 plays a key role in regulating the transition of cells from the G1 phase to the S phase and is encoded by the CCND1 gene. The expression of cyclin D1 protein is regulated at the transcription level, so CCND1 gene expression levels determine the progression of the cell cycle [26]. GI-761 at a concentration of 4 nM caused a 2-3-fold decrease in CCND1 in 24 hours.

Derivative VI-419-P3K at a concentration up to 300 *μ*g/ml has no effect on the number of double-strand DNA breaks and oxidative DNA damage in HELFs.

## 4. Conclusion

The antioxidant action of fullerene C60 and C70 derivatives is determined by their chemical structure. The difference in the chemical moieties attached to the fullerene core and the difference in the shape and size of the C60 and C70 cages lead to different cell responses to the derivatives. Antioxidative response master regulator NRF2 plays a key role in the determination of the prolonged antioxidant effect of fullerene derivatives on HELFs: activation of this transcription factor downregulates ROS production in cells. Derivative VI-419-P3K causes activation of NRF2 transcription both at gene and protein levels and induces translocation of NRF2 to the nucleus. This enables VI-419-P3K to have a prolonged antioxidant activity.

GI-761 does not increase the NRF2 expression, and the factor does not translocate to the nucleus. Because of this, GI-761 causes a secondary response of the cells that consists of an increased NOX4 expression accompanied by ROS production that leads to DNA damage. GI-761 could only potentially be used as a short-time antioxidant. The antioxidant action of GI-761 could be prolonged either by NOX4 inhibition or by NRF2 activation.

## Data Availability

The data used to support the findings of this study are included within the article.

## Supplementary Materials

Supplementary Materials Data concerning the method by which the compounds were obtained as well as the results of their 1H and 13C NMR and LS MS analysis.
