# Supplementary material for: Antioxidant Properties of Fullerene Derivatives Depend on Their Chemical Structure: A Study of Two Fullerene Derivatives on HELFs
Source: Oxid Med Cell Longev. 2019 Jan 17;2019:4398695. doi: 10.1155/2019/4398695 (PMC6360044; doi:10.1155/2019/4398695)
Supplement: Supplementary Materials — Data concerning the method by which the compounds were obtained as well as the results of their 1H and 13C NMR and LS MS analysis. [file 4398695.f1.docx]

**Supporting Information**

**Synthesis and characterization of the water-soluble fullerene derivatives**

**Compound 1.** Chlorofullerene C_60_Cl_6_ (100 mg, 0.107 mmol) was dissolved in toluene (100 mL) at room temperature. Anhydrous potassium carbonate (*ca.* 1g) and 4-amino-3-phenylbutanoic acid tert-butyl ester (151 mg, 0.643 mmol) were added and the reaction mixture was stirred for another 30 minutes. The reaction was controlled by TLC or HPLC. When the starting chlorofullerene has disappeared and a single major peak of the reaction product evolved, the mixture was filtered through a tight paper filter to separate potassium carbonate. The resulting solution was concentrated using a rotary evaporator to afford a reddish residue, which was washed with acetonitrile (30 mL) and dried in air. Compound **1**-O^t^Bu was obtained in 81% isolated yield.

Tert-butyl protecting groups of **1**-O^t^Bu were removed using trifluoroacetic acid. The mixture of **1**-O^t^Bu (161 mg, 0.085 mmol) and dichloromethane (20 mL) was strirred for 10 minutes until complete dissolution. Trifluoroacetic acid (969 mg, 8.5 mmol, 650 µL) was added and the reaction mixture was stirred for another 1h. Dichloromethane and trifluoroacetic acid were removed using a rotary evaporator to afford reddish powder, which was washed with acetonitrile (30 mL) and dried in air. Compound **1**-OH was obtained in 95% yield (130 mg).

To obtain water-soluble form of the fullerene derivative, mixture of compound **1**-OH (130 mg, 0.081 mmol), distilled water (10 mL) and anhydrous potassium carbonate (27.9 mg, 0.203 mmol) were stirred until complete dissolution. The transparent solution was filtered through a PES syringe filter (average pore size 0.45 μm) and freeze-dried for 5 h to afford reddish brown powder **1** with 95% isolated yield (138 mg).

**1-O^t^Bu.** ^1^H NMR (500 MHz, CDCl_3_, δ, ppm): 1.03 – 1.55 (m, 45H), 2.03 – 3.33 (m, 20H), 3.38 – 4.05 (m, 5H), 6.86 – 7.71 (m, 25H).

^13^C NMR (126 MHz, CDCl_3_, δ, ppm): 27.92, 27.98, 39.95, 40.26, 40.42, 42.99, 43.09, 43.12, 51.75, 51.84, 51.95, 80.29, 80.32, 80.37, 127.08, 127.73, 127.78, 127.92, 127.96, 128.06, 128.67, 129.21, 136.07, 138.91, 140.02, 140.89, 141.31, 141.79, 142.37, 142.83, 143.02, 143.32, 143.60, 143.89, 144.01, 144.12, 144.49, 144.95, 145.18, 147.12, 147.17, 147.24, 147.28, 147.33, 147.35, 147.99, 148.01, 148.36, 148.49, 148.52, 148.55, 148.64, 149.14, 149.30, 171.33, 171.43, 171.48.

**Compound 2.** A triple-neck round-bottom 50 mL flask equipped with a magnetic stirring bar, thermometer (0-150^o^C), stopper and condenser was evacuated and filled with argon 3 times. Chlorofullerene C_70_Cl_8_ (100 mg, 0.089 mmol) and 15-20 mL of dry nitrobenzene were introduced into the flask in a stream of argon. Methyl ester of 2-(3-phenylpropyl)malonic acid (2.224 g, 8.89 mol) and FeCl_3_ (10 mg) were added in a flow of argon. Reaction mixture was heated to 80-90^o^C and stirred for 0.5-1h, until TLC analysis showed complete disappearance of the pristine C_70_Cl_8_. Reaction mixture was cooled to the room temperature. Toluene (200 mL) was added and the reaction mixture was poured out on the top of the silica gel column. The target product was eluted using toluene/methanol mixtures (97-90%:3-10% v/v). Obtained solution was concentrated at the rotary evaporator, washed with acetonitrile or hexane and dried in air. Compound **2**-OMe was obtained as a dark-orange powder in 32% yield (81 mg).

Methyl ester protecting groups were removed by acidic hydrolysis. Mixture of compound **2**-OMe (0.028 mmol), toluene (25 mL), trifluoroacetic acid (5 mL), acetic acid (25 mL) and HCl (5 mL) was stirred at 65-70^o^C for 3-4 days and concentrated using a rotary evaporator to afford a dark-orange powder, which was washed with acetonitrile and dried in air. Compound **2**-OH was obtained in 95% yield (69 mg).

To obtain water-soluble form of the fullerene derivative, mixture of compound **2**-OH (69 mg, 0.027 mmol), distilled water (10 mL) and anhydrous potassium carbonate (29.2 mg, 0.212 mmol) were stirred until complete dissolution. The formed transparent solution was filtered through a PES syringe filter (average pore size 0.45 μm) and freeze-dried for 5 h to afford a dark orange powder **2** with 95% isolated yield (82 mg).

**2-OMe.** ^1^H NMR (500 MHz, CDCl_3_, δ, ppm): 1.48 – 1.78 (m, 16H), 1.87– 2.13 (m, 16H), 2.45 – 2.75 (m, 16H), 3.34 – 3.52 (m, 8H), 3.70 – 3.86 (m, 48H), 6.78 (d, 4H, *J* = 8.4 Hz), 6.83 (d, 4H, *J* = 8.3 Hz), 6.86 (d, 4H, *J* = 8.4 Hz), 7.09 (d, 4H, *J* = 8.3 Hz), 7.33 (d, 4H, *J* = 8.3 Hz), 7.39 (d, 4H, *J* = 8.3 Hz), 7.46 (d, 4H, *J* = 8.3 Hz), 7.71 (d, 4H, *J* = 8.2 Hz).

^13^C NMR (126 MHz, CDCl_3_, δ, ppm): 28.46, 28.50, 28.54, 29.33, 29.41, 34.96, 35.13, 51.61, 51.64, 51.66, 51.69, 52.60, 52.62, 52.64, 60.62, 60.81, 61.09, 61.69, 128.04, 128.09, 128.18, 128.22, 128.26, 128.61, 128.77, 132.01, 132.72, 134.34, 136.29, 136.31, 136.42, 136.55, 136.91, 137.99, 138.05, 140.30, 140.38, 140.47, 140.94, 142.51, 142.64, 143.31, 143.57, 145.32, 145.70, 146.00, 146.55, 146.79, 146.82, 148.20, 148.99, 149.72, 150.43, 150.47, 150.53, 152.37, 152.72, 152.89, 152.96, 153.71, 154.08, 154.24, 154.26, 154.81, 155.06, 155.37, 162.41, 169.85, 169.87, 169.90, 169.91.

**2-OH**. ESI MS: 1304 ([M]^2-^).
